# The genome sequence of the silver Y moth, *Autographa gamma *(Linnaeus, 1758)

**DOI:** 10.12688/wellcomeopenres.17758.1

**Published:** 2022-03-18

**Authors:** Douglas Boyes, Peter W.H. Holland, University of Oxford and Wytham Woods Genome Acquisition Lab, Darwin Tree of Life Barcoding collective, Wellcome Sanger Institute Tree of Life programme, Wellcome Sanger Institute Scientific Operations: DNA Pipelines collective, Tree of Life Core Informatics collective, Darwin Tree of Life Consortium

**Affiliations:** 1 UK Centre for Ecology and Hydrology, Wallingford, Oxfordshire, UK; 2 Department of Zoology, University of Oxford, Oxford, UK

**Keywords:** Autographa gamma, silver Y, genome sequence, chromosomal, Lepidoptera

## Abstract

We present a genome assembly from an individual female *Autographa gamma* (the silver Y; Arthropoda; Insecta; Lepidoptera; Noctuidae). The genome sequence is 373 megabases in span. The majority of the assembly (99.65%) is scaffolded into 32 chromosomal pseudomolecules, with the W and Z sex chromosomes assembled. The mitochondrial genome was also assembled and is 15.2 kilobases in length.

## Species taxonomy

Eukaryota; Metazoa; Ecdysozoa; Arthropoda; Hexapoda; Insecta; Pterygota; Neoptera; Endopterygota; Lepidoptera; Glossata; Ditrysia; Noctuoidea; Noctuidae; Plusiinae; Autographa; *Autographa gamma* (Linnaeus, 1758) (NCBI:txid254363).

## Background

*Autographa gamma* (silver Y) is a widespread and common noctuid moth found across the Palaearctic from Europe to Japan, and also in North Africa. The adult moth is commonly seen visiting nectar-rich flowers at dusk or in the daytime in late summer; the species also flies at night and is attracted to light. *A. gamma* is resident through winter only in the southern part of its range, but is highly migratory with large numbers of individuals moving north across Europe during spring and summer.

The impressive long distance migration is achieved through moths selecting fast-moving airstreams 200–1000 metres above ground, coupled with an internal compass sense so that flight is restricted to nights with favourable winds ( Chapman *et al*., 2008; Chapman *et al*., 2010). The migrating adults breed in high-latitude areas such as the UK to boost numbers further. Adults fly south in autumn to overwinter in southern Europe. Use of vertical-facing entomological radar coupled with insect trapping has revealed that the number of *A. gamma* adults reaching the UK varies from 10 million to over 200 million adult moths each year ( Chapman *et al*., 2008). The sporadic occurrence of huge numbers often brings *A. gamma* to wider attention. For example, during the Euro 2016 football final in Paris, prolonged use of floodlights attracted clouds of *A. gamma* , with moths settling on Cristiano Ronaldo and other players while watched by a television audience of over 280 million.

The larvae of *A. gamma* feed a wide range of herbaceous plants including clover and nettle; the larvae can also cause damage to cultivated crops notably lettuce, sugar beet, tomatoes, peas and cabbage ( Carter, 1984; Golikhajeh *et al.*, 2016). Two female sex pheromone components have been identified ( Dunkelblum & Gothilf, 1983) with similarity to the pheromone blends of closely related noctuid moths ( Becher & Revadi, 2020). The availability of a genome sequence will aid future research into migratory behaviour, pheromone production and chemoreception.

The genome of *A. gamma* was sequenced as part of the Darwin Tree of Life Project, a collaborative effort to sequence all of the named eukaryotic species in the Atlantic Archipelago of Britain and Ireland. Here we present a chromosomally complete genome sequence for *A. gamma*, based on one specimen from Wytham Woods, Oxfordshire, UK.

## Genome sequence report

**Figure 1.**
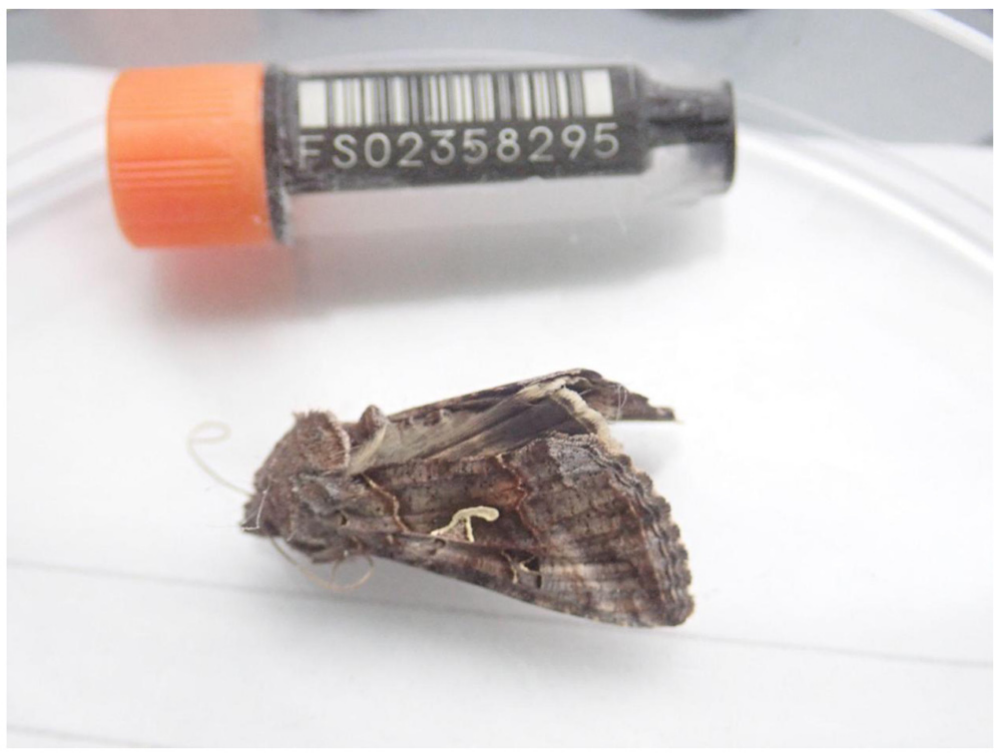
Image of the ilAutGamm1 specimen taken prior to preservation and processing.

The genome was sequenced from a single female *A. gamma* (ilAutGamm1; Figure 1) collected from Wytham Woods, Oxfordshire (biological vice-country: Berkshire), UK (latitude 51.775, longitude -1.332). A total of 36-fold coverage in Pacific Biosciences single-molecule long reads and 119-fold coverage in 10X Genomics read clouds were generated. Primary assembly contigs were scaffolded with chromosome conformation Hi-C data. Manual assembly curation corrected 20 missing/misjoins and removed 2 haplotypic duplications, reducing the assembly size by 0.06% and the scaffold number by 16.39%, and increasing the scaffold N50 by 6.60%. 

**Figure 2.**
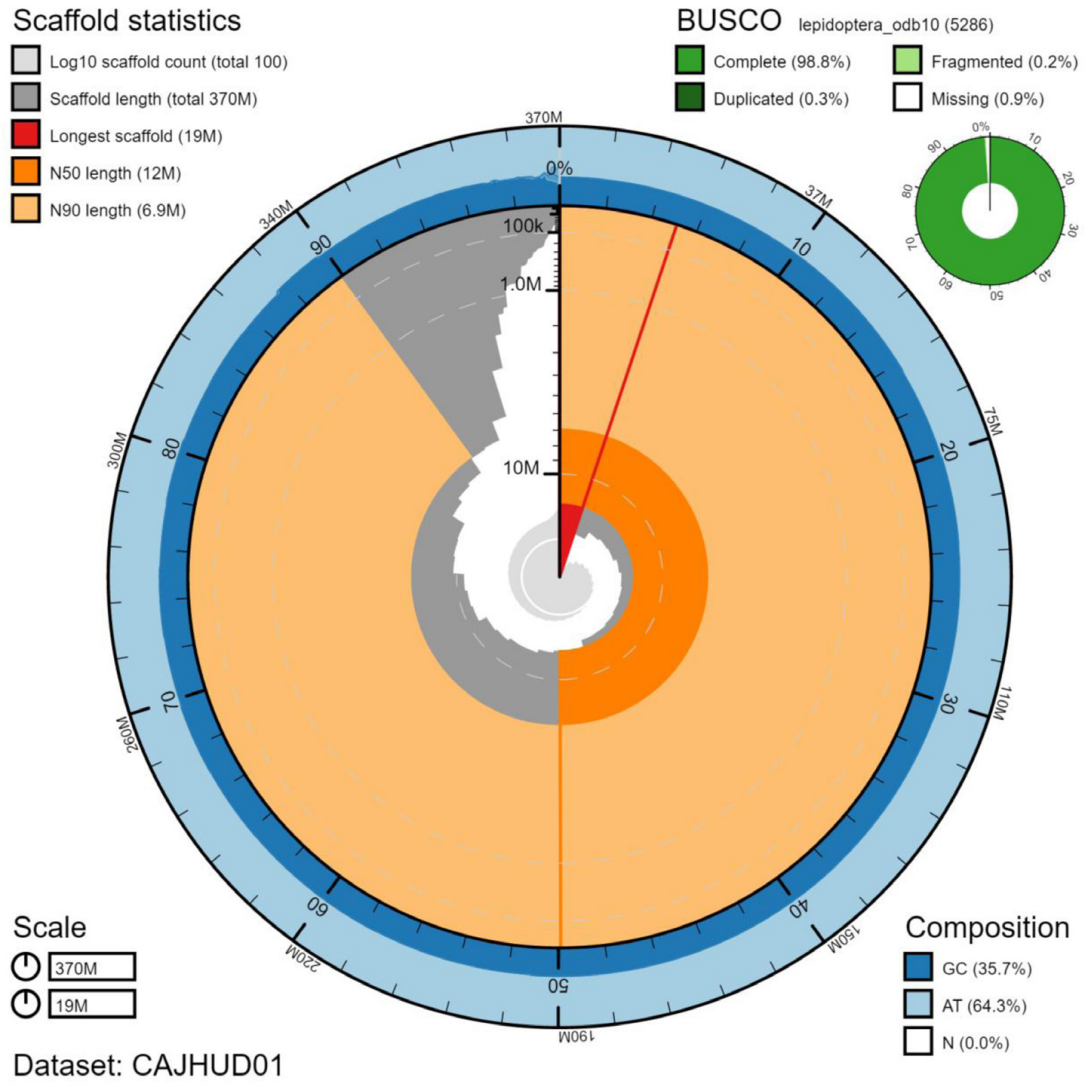
Genome assembly of *Autographa gamma*, ilAutGamm1.1: metrics. The BlobToolKit Snailplot shows N50 metrics and BUSCO gene completeness. The main plot is divided into 1,000 size-ordered bins around the circumference with each bin representing 0.1% of the 373,067,087 bp assembly. The distribution of chromosome lengths is shown in dark grey with the plot radius scaled to the longest chromosome present in the assembly (19,124,284 bp, shown in red). Orange and pale-orange arcs show the N50 and N90 chromosome lengths (12,167,202 and 6,891,251 bp), respectively. The pale grey spiral shows the cumulative chromosome count on a log scale with white scale lines showing successive orders of magnitude. The blue and pale-blue area around the outside of the plot shows the distribution of GC, AT and N percentages in the same bins as the inner plot. A summary of complete, fragmented, duplicated and missing BUSCO genes in the lepidoptera_odb10 set is shown in the top right. An interactive version of this figure is available at https://blobtoolkit.genomehubs.org/view/ilAutGamm1.1/dataset/CAJHUD01/snail.

The final assembly has a total length of 373 Mb in 102 sequence scaffolds with a scaffold N50 of 12.2 Mb ( Table 1). Of the assembly sequence, 99.65% was assigned to 32 chromosomal-level scaffolds, representing 30 autosomes (numbered by sequence length), and the W and Z sex chromosomes ( Figure 2– Figure 4; Table 2). The W chromosome is highly fragmented. The assembly has a BUSCO ( Manni *et al*., 2021) completeness of 98.8% (single 98.6%, duplicated 0.3%) using the lepidoptera_odb10 reference set (n=5286). While not fully phased, the assembly deposited is of one haplotype. Contigs corresponding to the second haplotype have also been deposited.

**Table 1.** Genome data for *Autographa gamma*, ilAutGamm1.1.

*Project accession data*	
Assembly identifier	ilAutGamm1.1
Species	*Phlogophora meticulosa*
Specimen	ilAutGamm1
NCBI taxonomy ID	NCBI:txid254363
BioProject	PRJEB42116
BioSample ID	SAMEA7519848
Isolate information	ilAutGamm1: female, head/thorax (genome assembly, RNA-Seq), abdomen (Hi-C); ilAutGamm2: unknown sex, abdomen (RNA-Seq)
*Raw data accessions*	
PacificBiosciences SEQUEL II	ERR6548401
10X Genomics Illumina	ERR6002586-ERR6002589
Hi-C Illumina	ERR6002590, ERR6002591
PolyA RNA-Seq Illumina	ERR6286703, ERR6787412
*Genome assembly*	
Assembly accession	GCA_905146925.1
*Accession of alternate haplotype*	GCA_905146835.1
Span (Mb)	373
Number of contigs	129
Contig N50 length (Mb)	11.2
Number of scaffolds	102
Scaffold N50 length (Mb)	12.2
Longest scaffold (Mb)	14.7
BUSCO* genome score	C:98.8%[S:98.6%,D:0.3%],F:0.2%,M:0.9%,n:5286

*BUSCO scores based on the lepidopetra_odb10 BUSCO set using v5.1.2. C= complete [S= single copy, D=duplicated], F=fragmented, M=missing, n=number of orthologues in comparison. A full set of BUSCO scores is available at https://blobtoolkit.genomehubs.org/view/ilAutGamm1.1/dataset/CAJHUD01/busco.

**Figure 3.**
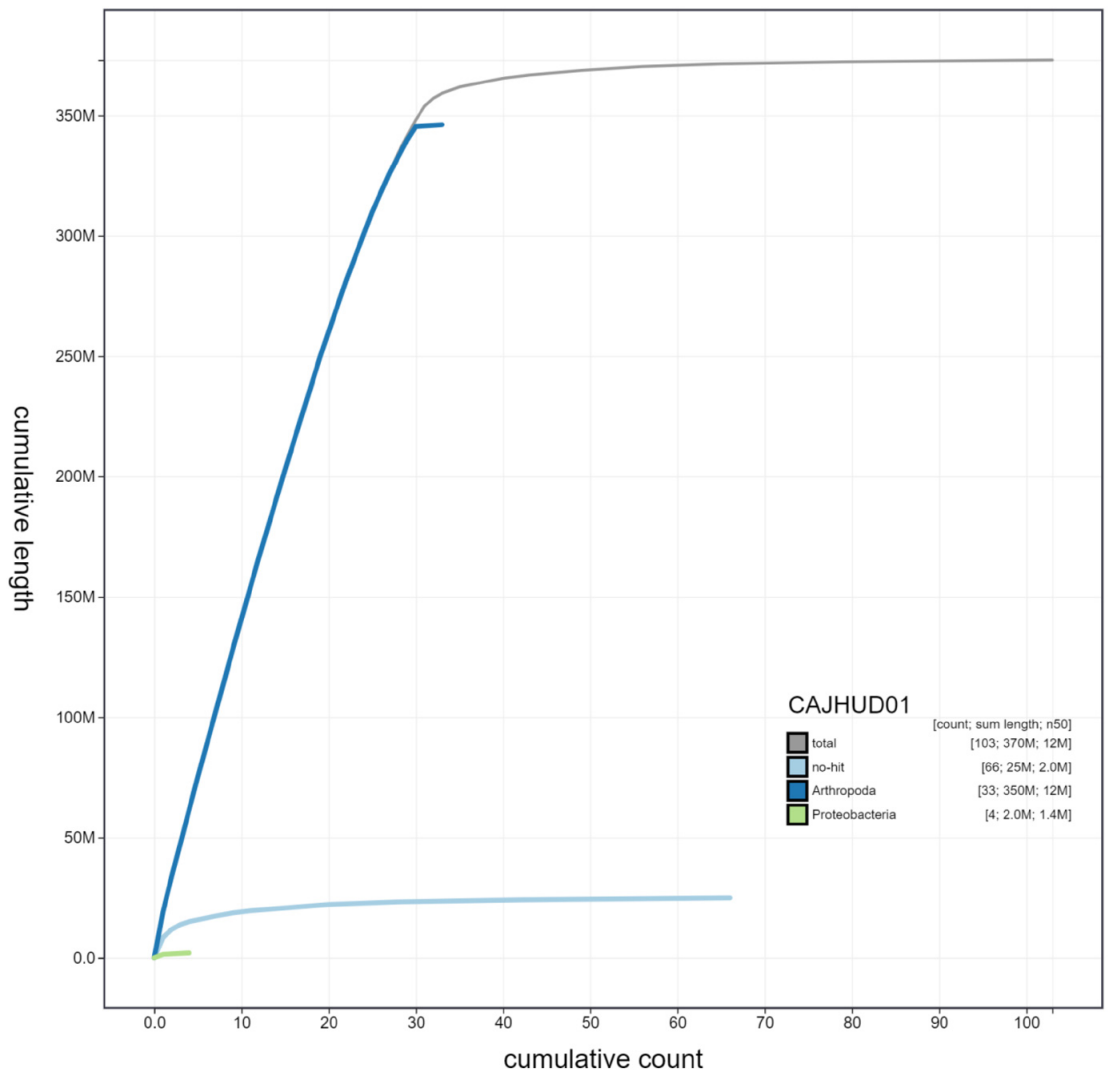
Genome assembly of *Autographa gamma*, ilAutGamm1.1: cumulative sequence. BlobToolKit cumulative sequence plot. The grey line shows cumulative length for all scaffolds. Coloured lines show cumulative lengths of scaffolds assigned to each phylum using the buscogenes taxrule. An interactive version of this figure is available at https://blobtoolkit.genomehubs.org/view/ilAutGamm1.1/dataset/CAJHUD01/cumulative.

**Figure 4.**
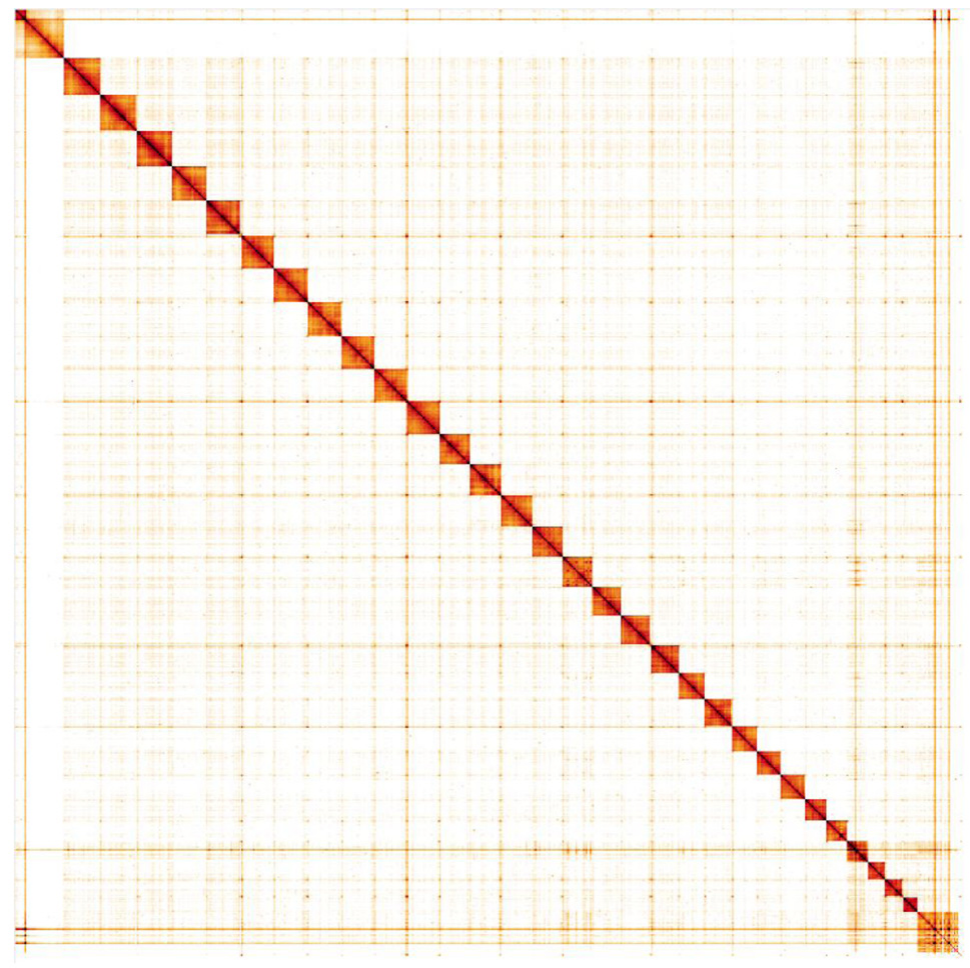
Genome assembly of *Autographa gamma*, ilAutGamm1.1: Hi-C contact map. Hi-C contact map of the ilAutGamm1.1 assembly, visualised in HiGlass.

**Table 2.** Chromosomal pseudomolecules in the genome assembly of *Autographa gamma*, ilAutGamm1.1.

INSDC accession	Chromosome	Size (Mb)	GC%
LR989850.1	1	14.66	35.8
LR989851.1	2	13.96	35.9
LR989852.1	3	13.80	36.0
LR989853.1	4	13.71	35.0
LR989854.1	5	13.41	35.9
LR989855.1	6	13.21	35.4
LR989856.1	7	13.11	35.2
LR989857.1	8	13.03	34.8
LR989858.1	9	12.89	35.0
LR989859.1	10	12.78	35.3
LR989860.1	11	12.76	35.4
LR989861.1	12	12.18	35.4
LR989862.1	13	12.17	35.6
LR989863.1	14	12.03	34.9
LR989864.1	15	11.79	35.0
LR989865.1	16	11.78	35.5
LR989866.1	17	11.55	35.3
LR989867.1	18	11.41	35.6
LR989868.1	19	10.76	36.1
LR989869.1	20	10.65	35.1
LR989870.1	21	10.47	35.8
LR989871.1	22	9.66	35.0
LR989872.1	23	9.56	35.9
LR989873.1	24	9.48	34.9
LR989874.1	25	8.49	35.4
LR989875.1	26	8.14	34.8
LR989876.1	27	7.98	38.1
LR989877.1	28	6.89	36.1
LR989878.1	29	6.78	36.7
LR989879.1	30	5.62	37.4
LR989880.1	W	3.29	37.8
LR989849.1	Z	19.12	35.9
LR989881.1	MT	0.02	19.9
-	Unplaced	15.89	37.4

## Methods

### Sample acquisition and nucleic acid extraction

One female *A. gamma* (ilAutGamm1) and one *A. gamma* of unknown sex (ilAutGamm2) were collected from Wytham Woods, Oxfordshire, UK (latitude 51.772, longitude -1.338) by Douglas Boyes, UKCEH, from woodland using a light trap. The specimen was identified by the same individual and preserved on dry ice.

DNA was extracted from head/thorax tissue at the Wellcome Sanger Institute (WSI) Scientific Operations core from the whole organism using the Qiagen MagAttract HMW DNA kit, according to the manufacturer’s instructions. RNA was extracted from abdomen tissue of ilAutGamm1 and ilAutGamm2 in the Tree of Life Laboratory at the WSI using TRIzol (Invitrogen), according to the manufacturer’s instructions. RNA was then eluted in 50 μl RNAse-free water and its concentration RNA assessed using a Nanodrop spectrophotometer and Qubit Fluorometer using the Qubit RNA Broad-Range (BR) Assay kit. Analysis of the integrity of the RNA was done using Agilent RNA 6000 Pico Kit and Eukaryotic Total RNA assay.

### Sequencing

Pacific Biosciences HiFi circular consensus and 10X Genomics Chromium read cloud sequencing libraries were constructed according to the manufacturers’ instructions. Poly(A) RNA-Seq libraries were constructed using the NEB Ultra II RNA Library Prep kit. Sequencing was performed by the Scientific Operations core at the Wellcome Sanger Institute on Pacific Biosciences SEQUEL II (HiFi), Illumina HiSeq X (10X) and Illumina HiSeq 4000 (RNA-Seq) instruments. Hi-C data were generated from abdomen tissue of ilAutGamm1 using the Qiagen EpiTect Hi-C kit and sequenced on HiSeq X.

### Genome assembly

Assembly was carried out with HiCanu ( Nurk *et al*., 2020); haplotypic duplication was identified and removed with purge_dups ( Guan *et al*., 2020). One round of polishing was performed by aligning 10X Genomics read data to the assembly with longranger align, calling variants with freebayes ( Garrison & Marth, 2012). The assembly was then scaffolded with Hi-C data ( Rao *et al*., 2014) using SALSA2 ( Ghurye *et al*., 2019). The assembly was checked for contamination and corrected using the gEVAL system ( Chow *et al*., 2016) as described previously ( Howe *et al*., 2021). Manual curation ( Howe *et al*., 2021) was performed using gEVAL, HiGlass ( Kerpedjiev *et al*., 2018) and Pretext. The mitochondrial genome was assembled using MitoHiFi ( Uliano-Silva *et al*., 2021), which performed annotation using MitoFinder ( Allio *et al*., 2020). The genome was analysed and BUSCO scores generated within the BlobToolKit environment ( Challis *et al*., 2020). Table 3 contains a list of all software tool versions used, where appropriate.

**Table 3.** Software tools used.

Software tool	Version	Source
HiCanu	2.1	Nurk *et al*., 2020
purge_dups	1.2.3	Guan *et al*., 2020
SALSA2	2.2	Ghurye *et al*., 2019
longranger align	2.2.2	https://support.10xgenomics.com/genome-exome/software/pipelines/latest/advanced/other-pipelines
freebayes	1.3.1-17-gaa2ace8	Garrison & Marth, 2012
MitoHiFi	1.0	Uliano-Silva *et al*., 2021
gEVAL	0.1.x	Chow *et al*., 2016
HiGlass	1.11.6	Kerpedjiev *et al*., 2018
BlobToolKit	2.6.2	Challis *et al*., 2020

### Ethics/compliance issues

The materials that have contributed to this genome note have been supplied by a Darwin Tree of Life Partner. The submission of materials by a Darwin Tree of Life Partner is subject to the Darwin Tree of Life Project Sampling Code of Practice. By agreeing with and signing up to the Sampling Code of Practice, the Darwin Tree of Life Partner agrees they will meet the legal and ethical requirements and standards set out within this document in respect of all samples acquired for, and supplied to, the Darwin Tree of Life Project. Each transfer of samples is further undertaken according to a Research Collaboration Agreement or Material Transfer Agreement entered into by the Darwin Tree of Life Partner, Genome Research Limited (operating as the Wellcome Sanger Institute), and in some circumstances other Darwin Tree of Life collaborators.

## Data availability

European Nucleotide Archive: Autographa gamma (silver Y). Accession number PRJEB42116; https://identifiers.org/ena.embl/PRJEB42116.

The genome sequence is released openly for reuse. The *A. gamma* genome sequencing initiative is part of the Darwin Tree of Life (DToL) project. All raw sequence data and the assembly have been deposited in INSDC databases. The genome will be annotated using the RNA-Seq data and presented through the Ensembl pipeline at the European Bioinformatics Institute. Raw data and assembly accession identifiers are reported in Table 1.

## Author information

Members of the University of Oxford and Wytham Woods Genome Acquisition Lab are listed here: https://doi.org/10.5281/zenodo.5746938.

Members of the Darwin Tree of Life Barcoding collective are listed here: https://doi.org/10.5281/zenodo.5744972.

Members of the Wellcome Sanger Institute Tree of Life programme are listed here: https://doi.org/10.5281/zenodo.6125027.

Members of Wellcome Sanger Institute Scientific Operations: DNA Pipelines collective are listed here: https://doi.org/10.5281/zenodo.5746904.

Members of the Tree of Life Core Informatics collective are listed here: https://doi.org/10.5281/zenodo.6125046.

Members of the Darwin Tree of Life Consortium are listed here: https://doi.org/10.5281/zenodo.5638618.
