# Supplementary material for: The genome sequence of the silver Y moth, Autographa gamma (Linnaeus, 1758)
Source: Wellcome Open Res. 2022 Mar 18;7:100. [Version 1] doi: 10.12688/wellcomeopenres.17758.1 (PMC11128053; doi:10.12688/wellcomeopenres.17758.1)
Supplement: Peer review history

# Reviewer report: Reviewer response for version 1

**DOI:** 10.21956/wellcomeopenres.19654.r70696

**Published:** 2024-05-25

**Authors:** Muzafar Riyaz

**Affiliations:** 1 Xavier Research Foundation, St Xavier's College, Palayamkottai, Tamilnadu, India

The article titled "The Genome Sequence of the Silver Y Moth, Autographa gamma (Linnaeus, 1758)" provides a comprehensive overview of the genome assembly of Autographa gamma, a widespread and common noctuid moth. The detailed assembly, sequencing methodology, and insights into the species' behavior contribute to both scientific knowledge and potential applications in agriculture and pest management.

Are sufficient details of methods and materials provided to allow replication by others?

Yes

Is the rationale for creating the dataset(s) clearly described?

Yes

Are the datasets clearly presented in a useable and accessible format?

Yes

Are the protocols appropriate and is the work technically sound?

Yes

Reviewer Expertise:

Molecular Phylogenetics, genomics, Phylogenomics

I confirm that I have read this submission and believe that I have an appropriate level of expertise to confirm that it is of an acceptable scientific standard.

# Reviewer report: Reviewer response for version 1

**DOI:** 10.21956/wellcomeopenres.19654.r70689

**Published:** 2024-05-25

**Authors:** Yan-Qun Liu

**Affiliations:** 1 Shenyang Agricultural University, Shenyang, Liaoning, China

This paper reported a chromosomal-level genome assembly from *Autographa gamma, *a widespread and common Noctuidae moth, that is helpful for understanding the genetic information of this species. Two minor revisions should be addressed.

 1. It is better to add the information of scaffold numbers for each pseudomolecule.  . 

 2. Species in Table 1. *Phlogophora meticulosa = Autographa gamma.*

Are sufficient details of methods and materials provided to allow replication by others?

Yes

Is the rationale for creating the dataset(s) clearly described?

Yes

Are the datasets clearly presented in a useable and accessible format?

Yes

Are the protocols appropriate and is the work technically sound?

Yes

Reviewer Expertise:

Insect Genetics and Genomics

I confirm that I have read this submission and believe that I have an appropriate level of expertise to confirm that it is of an acceptable scientific standard.

# Reviewer report: Reviewer response for version 1

**DOI:** 10.21956/wellcomeopenres.19654.r52002

**Published:** 2022-09-09

**Authors:** Li-Jun Cao

**Affiliations:** 1 Institute of Plant and Environmental Protection, Beijing Academy of Agriculture and Forestry Sciences, Beijing, China

Authors report the high quality genome assembly of the silver Y moth. The assembly has 32 chromosomal level scaffolds including both W and Z chromosomes. This high quality assembly will benefit future studies of the silver Y moth. W chromosome is rarely assembled in other Lepidoptera genomes. However, methods for identifying sex chromosomes have not been described.

Are sufficient details of methods and materials provided to allow replication by others?

Yes

Is the rationale for creating the dataset(s) clearly described?

Yes

Are the datasets clearly presented in a useable and accessible format?

Yes

Are the protocols appropriate and is the work technically sound?

Yes

Reviewer Expertise:

Molecular ecology, population genetics, population genomics

I confirm that I have read this submission and believe that I have an appropriate level of expertise to confirm that it is of an acceptable scientific standard.
